# Apps to Support Self-Management for People With Hypertension: Content Analysis

**DOI:** 10.2196/13257

**Published:** 2019-06-03

**Authors:** Chi Yan Hui, Emily Creamer, Hilary Pinnock, Brian McKinstry

**Affiliations:** 1 Asthma UK Centre for Applied Research, Usher Institute of Population Health Sciences and Informatics The University of Edinburgh Edinburgh United Kingdom; 2 Usher Institute of Population Health Sciences and Informatics The University of Edinburgh Edinburgh United Kingdom

**Keywords:** hypertension, self-management, telehealth, telemedicine, mobile app

## Abstract

**Background:**

Home blood pressure monitoring (HBPM) is one component of effective supported self-management, which may potentially be mediated by mobile apps.

**Objective:**

The aim of this study was to identify the self-management features (HBPM and broader support strategies) offered by currently available apps and to determine the features associated with download frequency and user ratings.

**Methods:**

We searched Google Play store, Apple App store, National Health Services Apps Library and myhealthapps.net (first search on February 1, 2018; updated August 18, 2018). We included high blood pressure apps available in the United Kingdom and extracted their features, number of downloads, and the average users’ rating from the app stores. We mapped the features to the holistic Practical Reviews In Self-Management Support (PRISMS) taxonomy of self-management support. We employed a regression analysis to determine if any features were associated with download frequency or user rating.

**Results:**

We included 151 apps. The 3 most common features were as follows: monitoring blood pressure (BP) and charting logs; lifestyle (exercise or dietary) advice; and providing information about hypertension. The other 11 components of the PRISMS taxonomy were rarely featured. There was little evidence to support associations between specific features and the download statistics and rating scores, with only 2 uncommon features achieving borderline significant associations. The presence of social support features, such as a forum, was weakly but significantly (*R*^2^=.04, *P*=.02) correlated with the number of downloads. Apps designed specifically for particular BP monitors/smart watches were weakly associated with a higher rating score (*R*^2^=.05, *P*<.001). Apps with more ratings were associated with more downloads (*R*^2^=.91, *P*<.001).

**Conclusions:**

The functionality of currently available apps is limited to logging BP, offering lifestyle advice, and providing information about hypertension. Future app development should consider broadening the remit to produce a system that can respond flexibly to the diversity of support that enables people to self-manage their hypertension.

## Introduction

High blood pressure (HBP) currently affects 1.13 billion people worldwide [1], and this is expected to rise to 1.5 billion by 2025 [2]. Globally, hypertension contributes to 9.4 million deaths annually [3], mainly from heart disease and stroke [4]. Identifying and controlling hypertension significantly reduces cardiovascular events [5]; yet, despite effective medication, blood pressure (BP) remains to be poorly controlled [6-8], at least in part owing to poor adherence by patients and reluctance to intensify medication by clinicians [9].

Supported self-management can improve control [10] with home BP monitoring (HBPM)—a widely used component of effective interventions. This not only avoids repeated visits to health care professionals for monitoring but also improves accuracy by allowing detection of *white-coat* and *masked* hypertension, which are common in both untreated and treated patients and result in misleading clinic BP readings [11]. HBPM can increase patient engagement in the management of their condition and provide a trusted basis for shared management decisions, including medication changes [12]. Furthermore, one mechanism by which HBPM works is by bridging the gap between patients’ perceptions of hypertension as a disease with multiple symptoms compared with the professional view of an asymptomatic condition, fostering confidence in the ability to self-manage [10]. There is strong evidence that when BP readings are sent electronically to and dealt with by clinicians, the outcomes are improved [13].

The development of affordable home BP monitors [14] and increasing ownership of smartphones among the general population [15] present opportunities for mobile apps to support patients’ self-management of their hypertension [16]. Many hypertension apps are available in the market [17]. However, there has been considerable concern voiced over the highly variable quality and appropriateness of these apps [18].

Self-monitoring, however, is only 1 component of supported self-management. The Practical Systematic Review of Self-Management Support (PRISMS) taxonomy describes a broader approach to supporting people to proactively self-manage their condition, offering a picklist of 14 potential support strategies associated with effective self-management interventions [19]. The purpose of this study was to map the landscape of hypertension apps that are currently available and assess the extent to which these apps deliver recognized components of self-management support (PRISMS) and whether there is a correlation between the app’s self-management features and their number of downloads and user ratings.

## Methods

### Search Strategy

We searched, identified, and screened apps on Google Play store [20], Apple iTunes preview website (using Google site search code of site:itunes.apple.com/gb/app), National Health Services (NHS) Apps Library [21], and *myhealthapps.net* [22] on February 1, 2018 (updated August 18, 2018), using the key term, *high blood pressure*. The search was restricted to the apps available in the United Kingdom.

### Screening and Data Extraction

We used the Google Web Scraper (version 0.3.7) [23] to extract the app name, description, number of raters, ratings, number of downloads (not available for iOS apps), version, latest updated date, cost, and the developers’ name. A reviewer (CYH) screened the extracted data using the following inclusion/exclusion criteria:

- *Inclusion criteria:* The app (1) advertised features relevant to management of hypertension, (2) was available for download through the official Android or Apple App stores/NHS Apps Library/myhealthapps.net, and (3) was written in English.
- *Exclusion criteria:* The app (1) could not be opened and/or used owing to technical problems after 3 attempts, (2) had not been updated within 1 year of the search date (ie, the last update was before February 1, 2016) to exclude those apps that were no longer being supported, (3) was designed for children, and (4) had been in the market for greater than 1 month but had no ratings and/or reviews to reduce the chance of including apps that were still under development.

A reviewer (CYH) reviewed the description, screenshots, and the developer website of the included apps and extracted their key app features using a piloted data extraction sheet under the headings of the 14 components of the PRISMS taxonomy (Textbox 1). Apps without clear information were downloaded and the features were assessed manually.

### Data Analysis

#### App Self-Management Features

We used the PRISMS taxonomy (see Textbox 1) [19] to categorize the extracted app features. We searched for the presence of each of these 14 items within each included app, dichotomizing the outcome as *present* (=1) or *absent* (=0). We counted the total number of apps addressing the 14 components of the taxonomy.

### Association Between the Practical Reviews in Self-Management Support Self-Management Components, Downloads, and Ratings

We performed a regression analysis in Microsoft Excel. We wanted to investigate the features [24] that may influence people to download and rate a BP app. Apps with apparently 500,000,000 or more recorded downloads were excluded in the regression analysis. We decided to adopt this approach to reduce the possibility of including apps that were no longer supported by developers. In addition, 1 app seemed to have such unlikely download statistics (exceeding popular apps, such as YouTube, TikTok, Pokemon GO, Snapchat [50,000,000 to 500,000,000 downloads]) that we decided to exclude it.

**Items included in the Practical Reviews in Self-Management Support (PRISMS) taxonomy of self-management support.**

- Information about condition and/or its management
- Information about available resources
- Provision of/agreement on specific clinical action plans and/or rescue medication
- Regular clinical review
- Monitoring of condition with feedback
- Practical support with adherence (medication or behavioral)
- Provision of equipment
- Provision of easy access to advice or support when needed
- Training/rehearsal to communicate with health care professionals
- Training/rehearsal for everyday activities
- Training/rehearsal for practical self-management activities
- Training/rehearsal for psychological strategies
- Social support
- Lifestyle advice and support

The number of downloads and the mean rating score were the outcome variables that we used in the regression analysis. The app’s information on the download page (rating, number of raters, cost, multiple conditions’ focus, the number of features, whether the app was developed by an internationally known company, presented as part of an established clinical program or claimed to be recommended by health care professionals, supported by a patient or professional organizations, or promoted by a campaign) were used as additional predictor variables as these features are known to influence downloads more generally [25]. Both outcome and predictor variables were standardized by using an Excel standardized function [26]. A simple linear regression analysis was performed to determine the relationship between independent variables and the downloads and ratings followed by multiple regression. We considered significance at the 5% level. All variables were entered into the multiple regression. The goodness-of-fit was assessed using R-squared statistics.

### Interpretation

We discussed the results of the data synthesis within a multidisciplinary team of 16 people, which included expertise in primary care (n=1), hypertension (n=2), and electronic health and digital technology (n=13). The features that were associated with the downloads and user rating score were of key interest to the multidisciplinary team and the relevance of particular aspects of the PRISMS taxonomy in relation to BP apps. There was considerable discussion about the relevance of metrics such as *number of reviewers* as a predictor of quality. The results were used to guide the design of a prototype hypertension app.

## Results

### Characteristics of Included Apps

**Figure 1:**
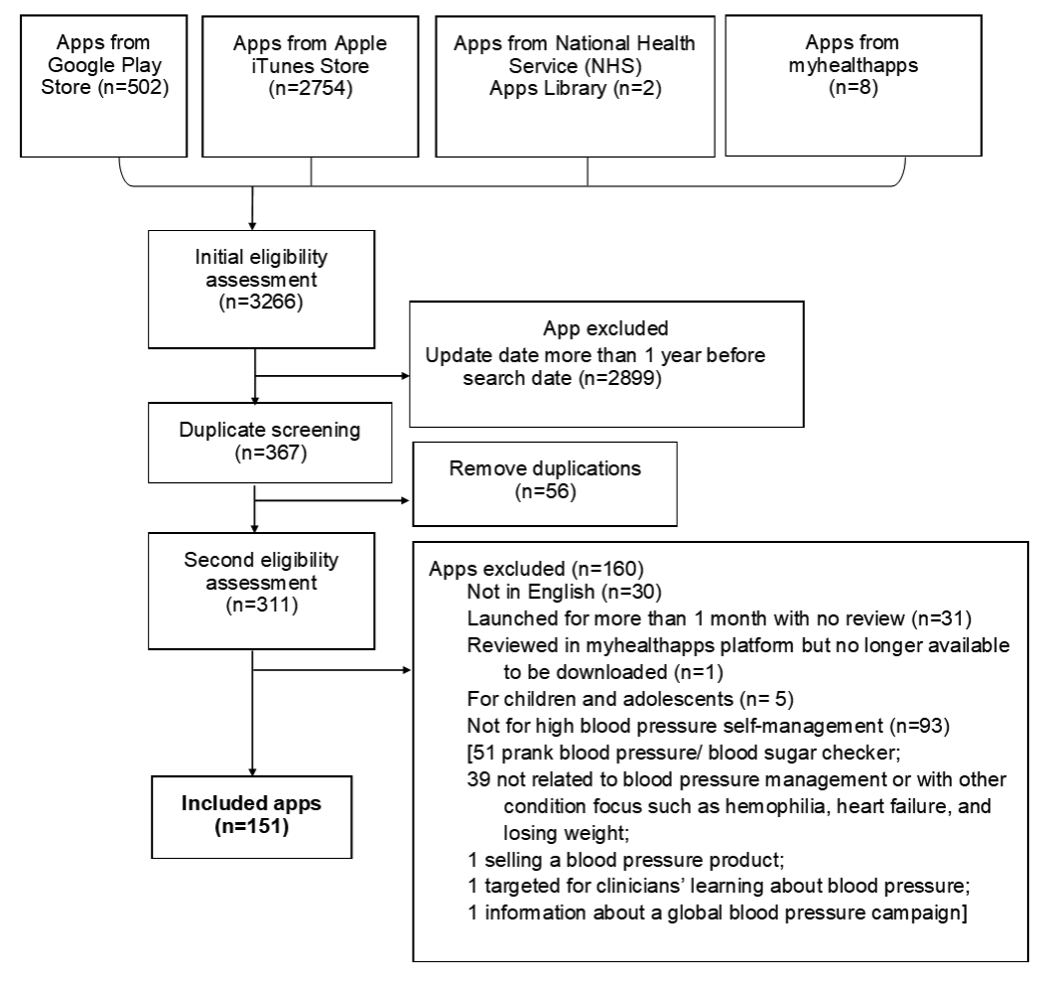
Flowchart of app selection and screening process.

A total of 151 apps were identified. Most were identified in Google Play and the Apple app store and a much smaller number from the NHS Apps Library and myhealthapp.net. The apps identified, the screening process, and the final numbers of the apps included are detailed in the flowchart (Figure 1).

Of the 151 apps, 95.4% (144/151) apps were available on both Google Play and Apple App store, 4.6% (7/151) apps were only available on the Apple App store [27-33]. Furthermore, 51.0% (77/151) apps focused solely on HBP and 47.7% (74/155) addressed multiple clinical conditions including diabetes, heart disease, respiratory disease, kidney disease, cancer, and psychological conditions. Also, 6.6% (10/151) apps were presented as part of an established clinical program, and 1 app advertised on the app store claimed that it was recommended by doctors and pharmacists. Only 5.2% (8/155) apps charged users (£0.9 to £7.90). Furthermore, 3 apps were supported by patient or professional organizations (the American Heart Association, The George Institute for Global Health, and American College of Cardiology Foundation); 1 app was designed for a campaign (Action for Happiness); 3.2% (5/155) apps were developed by BP monitor/smart watch companies (Samsung, Beurer, Braun, Omron, and Withings); 1 app was developed by an insurance company (AXA); and 28 apps were designed to be connected with a particular brand of BP monitor or smart watch.

### Apps Self-Management Features

Table 1 summarizes the application features related to the 14 components of the PRISMS taxonomy [19]. Details are shown in the Multimedia Appendix 1.

**Table 1:** Application features categorized by the Practical Reviews in Self-Management Support (PRISMS) taxonomy.

Taxonomy item and app features		Apps, n
**Information about condition and/or its management^**a**^**		
	News about blood pressure (BP), diet advice, what qualifies as high BP, home monitoring and diagnosis of BP, specific advice for BP during pregnancy	30
**Information about available resources**		
	Providing links to WebMD, American Heart Association, NHS^b^ information pages, and other associations	2
**Provision of/agreement on specific clinical action plans and/or rescue medication**		
	Action plan (eg, personalized agreement on what to do if BP is high/low)	1
**Regular clinical review**		
	Manual input of appointment details (eg, date and location)	2
	Booking appointments	3
**Monitoring of condition with feedback^**a**^**		
	Logging systolic/diastolic BP, heart rate, medication taken, and free-text notes	70
	Nonvalidated and likely misleading *measuring* of BP (via camera)	3
	Nonvalidated and likely misleading *measuring* of BP (via fingerprint)	3
	Voice assistant to support logging activities such as making and sending logs	3
	Sending logs (xls/db/PDF) via email; saved in Dropbox, Google Drive, SD card); share logs via portal	58
	Sending logs by using the app portal, SMS^c^, email, or via an automated telephone call; data transferred to GP^d^ systems such as Egton Medical Information Systems Web and SystemOne	2
	Showing logs on charts (pie chart, line chart) only	37
	Providing observations such as *your BP hasn't varied a lot in the last months*, *your BP is normal*, *you are stage 2 hypertension*.	8
	Providing simple advice and suggesting actions such as *condition: stage 2 hypertension; to do: you may have high blood pressure*, *change your lifestyle - see your doctor, aim for a total blood pressure less than 120/80 mmHg*, *avoid Tobacco, etc*, *hypertensive urgency - please go to the hospital!*, *your blood pressure is dangerously high. See your GP urgently or call 111 for more advice*.	7
	Showing logs on colored chart/bar/points to indicate hypertension (stage 1), hypertension, prehypertension, normal and low BP	30
	Showing the person’s BP at different times of the day in different places	1
	Showing the number of days that meet the preset optimized BP value	1
**Practical support with adherence (medication or behavioral)**		
	Medication reminder	11
	Points/rewards to encourage logging	5
**Provision of equipment**		
	Use the proprietary or Bluetooth smart meter to log systolic/diastolic/pulse/heart rate	19
	Connecting to activity tracker gadgets or smart watches	16
**Provision of easy access to advice or support when needed**		
	Web doctor consultation	1
	Communication via texting	1
**Training/rehearsal to communicate with health care professionals**		
	Personal health record accessible to health care professional to enhance discussions or providing patient access to health record	6
**Training/rehearsal for everyday activities**		
	Not applicable	0
**Training/rehearsal for practical self-management activities**		
	Tagging logs to help users to understand what causes their high BP (eg, add tag *before eating*)	3
	*Logging reminder* to train users to make regular logs for monitoring	10
	Entering personal health profile, preparing for emergency conditions	2
	Ordering repeat prescriptions	1
	Emergency call to GP	1
**Training/rehearsal for psychological strategies**		
	Using breathing exercises to relieve stress to lower BP	5
**Social support**		
	Forum to share stories or advice about BP management	3
**Lifestyle advice and support^**a**^**		
	Providing DASH^e^/low sodium diet advice/videos to lower BP, advice on exercise intensity, lifestyle factors that cause high BP, managing stress, breathing exercises, relaxing games to lower BP	54
	Importing and showing weight data and sleep duration from another app (*Weight Companion*, *Sleep as Android*, *MyFitnessPal*, *Google Fit*, *Samsung Health*)/other third-party apps	7
	Sending messages to users via WhatsApp and Skype	1
	Log exercise intensity, BMI, and diet - make a note of the measurements, which may be associated to the HBP^f^	10
	Activity tracking (bicycle/steps) via Fitbit alongside blood pressure monitoring	20

^a^Top *3* common BP self-management supports found *in* the apps.

^b^NHS: National Health Services.

^c^SMS: short message service.

^d^GP: general practitioner.

^e^DASH: Dietary Approaches to Stop Hypertension.

^f^HBP: high blood pressure.

#### Monitoring of Blood Pressure With Feedback

The most common self-management support provided by the apps was *monitoring of condition with feedback* (70/151, 46.4%), though only 1 UK app provided feedback integrated with current National Health Service (NHS) general practice systems such as Egton Medical Information Systems and SystemOne [34]. Monitoring involved logging systolic/diastolic BP and heart rate. Most apps (58/70, 83%) enabled sharing of logs with health care professionals, and all of them provided feedback/advice. Advice popped up immediately after people entered their BP reading as opposed to popping up when the BP breached a certain threshold. Most of the feedback took the form of simply displaying the user’s BP logs back to them in a chart format (37/70, 53%). However, 9 of the 70 apps (13%) provided an additional basic evaluation of the recorded BP value, such as *your BP is normal*, and 6 apps (9%) coupled this type of evaluative feedback with advice on actions to take, such as (to a patient with a BP of 160/110) *You are hypertensive Urgency [sic]—please go to the hospital!* Furthermore, 1 app showed the patient’s BP values at different times of the day and in different places [35]. In addition, 1 app showed the number of days that met the preset optimized BP value [36].

#### Lifestyle Advice and Support

The second most common category of self-management support was *Lifestyle advice and support* (54/151, 35.8%). Examples include DASH (Dietary Approaches to Stop Hypertension) [37] and suggestions for low-sodium cooking recipes to help lower BP. The recipes were not targeted to individuals, for example, general dietary advice was not provided in relation to the monitored weight data.

#### Information

The third most common self-management support was *Information about condition and/or its management* (30/151, 19.9%). The apps provided information such as news items about hypertension, diagnostic criteria for HBP, how to perform home monitoring, and specific advice for BP during pregnancy. Patients could choose what information they wanted to read and follow.

#### Other Less Common Features

One app that was tailored for patients with multiple conditions provided an action plan template on the app. The template was a free text template in which the patient should enter the personalized agreement with their clinician on what to do if BP was high or low and assigned a color (green, amber, and red) for the specific action. Furthermore, 1 app (incurring a charge) offered 24/7 Web consultation with a third-party health care specialist team. Another app allowed patients to text the specialist team for enquiries. None of the apps were found to provide everyday activity training support.

### Features Associated With Downloads

A total of 144 apps had the number of downloads displayed on the Google Play store. Of these, the number of downloads ranged from 1 to 500,000,000. These numbers provided snapshots on the included app’s adoption rate. Furthermore, 90.3% (130/144) apps had fewer than 500,000 downloads, 9.0% (13/144) apps had 500,000 to 50,000,000 downloads, 1 app was an outlier with more than 500,000,000 downloads and was excluded in the regression analysis. Moreover, 2 apps included a service in which health care professionals gave feedback to patients. The number of downloads was available for one of the apps that was available on the Google Play store, and it did not attract a high number of downloads (number of downloads=100). However, it is not clear how restricted the recruitment was for this app. There were no missing data in the analysis. The results of the simple linear regression analysis are shown in Table 2. In addition, 1 feature (the inclusion of social support features, such as a forum) was weakly correlated but achieved statistical significance (*R*^2^=.04, *P*=.02). Perhaps, unsurprisingly, the number of raters on the app stores (*R*^2^=.91, *P*<.001) was associated with the number of downloads. A multiple regression analysis showed significant impact from a combination of the variables (*R*^2^=.92, *P*<.001), of which *the number of raters* was the strongest contributor among the variables (coefficient=.96, *P*<.001). Other variables had coefficients of .01 or .001 with a *P* value that was more than .1. The details are shown in Multimedia Appendix 2.

### Features Associated With Average Rating

Of the 148 apps, the average rating ranged from 0 to 5 (out of 5). Furthermore, 113 ratings were from the Google Play store, 35 ratings were from the Apple app store. In addition, 14 apps had an average rating of 5, 12 of them were rated 0. These apps had been available on the app markets for less than a month. Moreover, 2 apps in which health care professionals gave feedback were not associated with high overall ratings (one scored 0 and another 2.5). There were no missing data in the regression analysis. There was little or no association between the presence of PRISMS features and the number of downloads.

**Table 2:** *R*^2^ and *P* values of a simple regression analysis at 5% significance level for downloads (Data were sorted by the *P* values in ascending order).

Variables		Standardized coefficient	*R* ^2^	*P* value
**PRISMS^a^ features**				
	Social support^b^	0.19	0.04	.02
	Information about condition and/or its management	0.13	0.02	.12
	Lifestyle advice and support	–0.08	0.01	.34
	Training/rehearsal for practical self-management activities	0.05	<0.01	.56
	Training/rehearsal for psychological strategies	–0.04	<0.01	.61
	Information about available resources	–0.03	<0.01	.72
	Regular clinical review	–0.03	<0.01	.76
	Practical support with adherence (medication or behavioral)	–0.03	<0.01	.76
	Training/rehearsal to communicate with health care professionals	–0.02	<0.01	.79
	Provision of equipment	–0.02	<0.01	.81
	Provision of/agreement on specific clinical action plans and/or rescue medication	–0.02	<0.01	.84
	Monitoring of condition with feedback	–0.01	<0.01	.87
	Provision of easy access to advice or support when needed	–0.01	<0.01	.91
	Training/rehearsal for everyday activities	N/A^c^	N/A	N/A
**Other possible associated factors**				
	Number of raters^b^	0.95	0.91	<.001
	Rating	0.06	<0.01	.48
	Paid	–0.05	<0.01	.57
	Supported by associations or apps for a campaign	–0.04	<0.01	.65
	Apps for multiple conditions	–0.03	<0.01	.72
	Recommended by health care professionals/use in the NHS practices	–0.02	<0.01	.8
	Numbers of features	0.01	<0.01	.89
	Created by internationally BP^d^ monitor/smart watch company	0.01	<0.01	.9

^a^PRISMS: Practical Reviews In Self-Management Support.

^b^The independent variables with *P* values <.05, which were assessed as statistically significant.

^c^N/A: not applicable.

^d^BP: blood pressure.

**Table 3:** *R*^2^ and *P* values of a simple regression analysis at 5% significance level for user ratings (Data were sorted by the *P* values in ascending order).

PRISMS^a^ self-management features	Standardized coefficient	*R* ^2^	*P* value
Provision of equipment^b^	–0.23	0.05	.01
Monitoring of condition with feedback	–0.16	0.02	.07
Information about condition and/or its management	0.14	0.02	.13
Training/rehearsal for psychological strategies	0.13	0.01	.15
Training/rehearsal to communicate with health care professionals	0.08	<0.01	.41
Social support	–0.07	<0.01	.42
Practical support with adherence (medication or behavioral)	–0.06	<0.01	.50
Provision of easy access to advice or support when needed	0.06	<0.01	.51
Training/rehearsal for practical self-management activities	0.04	<0.01	.64
Lifestyle advice and support	–0.03	<0.01	.72
Information about available resources	–0.03	<0.01	.73
Regular clinical review	0.02	<0.01	.83
Provision of/agreement on specific clinical action plans and/or rescue medication	0.01	<0.01	.87
Training/rehearsal for everyday activities	N/A^c^	N/A	N/A

^a^PRISMS: Practical Reviews in Self-Management Support.

^b^The independent variable with *P* values <.05, which were assessed as statistically significant.

^c^N/A: not applicable.

The linear regression analysis in Table 3 showed that apps designed specifically for particular BP monitors or smart watches were weakly associated with having higher user ratings (*R*^2^=.05, *P*<.001). Other PRISMS features [19] were not significantly correlated with better reviews. Multiple regression showed there was no significant association between a combination of the features (Multimedia Appendix 2).

## Discussion

### Principal Findings

Current BP apps focus on monitoring with generic feedback, lifestyle advice, and information about the condition. Monitoring and feedback typically involve charts and summary logs. Only 7 apps provide specific advice about what to do if the BP is high or low, and of these, only 1 provided advice based on a clinical action plan agreed with a health care professional. Lifestyle advice was mainly dietary advice, advising people to reduce sodium intake to lower BP, or advice on exercise intensity, weight loss, and stress management. None of them provided any evidence for these interventions. The other 11 components of the PRISMS taxonomy were rarely featured in the apps we reviewed.

There was no evidence to support associations between specific features and the *popularity* of the app as implied by download statistics and rating scores, with only 2 features achieving borderline significant associations. Apps with more raters were associated with more downloads; however, a more frequently downloaded app will have a much larger potential pool to provide reviewers, and therefore, the direction of causation is unclear.

### Strengths and Limitations

This study maps the app features onto the PRISMS taxonomy, a theoretically based list of components derived from a systematic metareview of 969 trials and 30 qualitative studies of self-management support interventions [19]. We initially performed a search in February 2018 but updated this in August 2018. Despite detecting a large number of new apps, some emerging apps may be missed in the study.

There are some methodological limitations. First, although we investigated the relationship between the app features and the number of downloads and the user rating in the app store, there are many other variables, such as the ranking in the search results in the app store or Google search engine or recommendations from personal physicians, friends, and relatives, which may also influence downloads and user ratings. Second, for resource reasons, the data extraction and review of the apps’ content was completed by 1 person. However, the data were extracted by machine code to reduce the risk of human error in data extraction. The results were discussed in a multidisciplinary team to provide a broader interpretation of the results. Third, the rating in the Google Play app store is the sum of the average rating in each version of the app, whereas the rating in the Apple app store is the sum of the app rating over the total number of raters. We did not adjust the user ratings in the regression analysis as the average rating of each version was not available directly on the Google Play store. A yearly follow-up data extraction is needed to calculate this information. However, our review gives a snapshot of the current BP apps in the market. Fourth, the number of downloads and users’ rating reflected user perceptions rather than clinical effectiveness or utility of the apps, nor did these measures provide information on the sustained use of those apps. Nevertheless, it provides a consumer's view to inform app developers about possible desirable features to be developed. Fifth, we excluded those apps without continued support from their developers as a yearly analysis is needed to review those apps. We think it is important to focus the review on those that have survived the market.

### Interpretation in Relation to the Published Literature

Little appears to have changed in the 2 years since Jamaladin [38] conducted the content analysis on BP apps in 2016. Logging of BP remains the most common feature in BP apps, with feedback limited to providing graphical records of logs. Research involving people with different long-term conditions shows that people want clinical and customized advice on what to do when their condition is getting worse (as published in Hui et al [39] and the study by Mendiola et al [17]). App developers’ reluctance to develop apps with customized advice may relate to concerns about potential medicolegal consequences and/or the additional regulatory burden involved in meeting medical device legislation requirements [40].

More remarkable is the very narrow focus of the BP apps. Our use of the PRISMS taxonomy highlighted how rarely most of the components of self-management support were provided by the apps we reviewed. The taxonomy is described as a *tick list* and not a *check list* as different components will be relevant in different clinical and social contexts and to people with different preferences. Nevertheless, it would seem that the technology market has not yet recognized the potential breadth of self-management support and produced an app that can flexibly provide a broad range of features to support people to live with their hypertension.

We found that a greater number of raters were associated with higher downloads. This may seem obvious as a higher number of users means that there are more consumers to rate an app. However, there is evidence that online shopping customers tend to purchase products with a large number of reviews [41]. Furthermore, there is evidence that users who do not engage with the qualitative detail of app reviews tend to be greatly influenced by the *quantity* of raters on the download page [25]. Downloads of apps linked to BP monitors and smart watches may be driven by sales of the peripherals rather than the app. In addition, the device is likely to be supported by a helpline, which may be a valuable feature for people less familiar with app technology—potentially the older patients with hypertension (Hui et al [39]).

### Conclusions

The number of apps for people with hypertension is increasing, but their functionality remains static, typically limited to logging BP, offering lifestyle advice, and providing information about hypertension. There is no clear evidence that these or any specific features of apps are associated with increased downloads or improved ratings.

We suggest app developers use an evidence-based framework such as PRISMS to ensure that their apps include the breadth of components that provide support for self-management. In addition, it is important to review the app’s usage iteratively and collect feedback from users (both patients and professionals) to continuously improve features. Ideally, apps should be easy to use, provide immediate feedback on the BP reading possibly related to a clinician-recommended action plan, warn of dangerous BPs, provide access to information about self-management of BP and how to measure it properly, allow users to opt for reminders to check BP and to take medications, and to choose to communicate readings with professionals.

The challenge for app developers is to look outside their comfort zone of features that are technologically straightforward and to produce a flexible app that connects with health/social/community services to enable it to respond to the known breadth and diversity of support that will enable people to live with and self-manage their hypertension.

Devices that provide advice based on received readings are classed as medical devices and must pass stringent and expensive evaluations to satisfy the Medicines and Health care Products Regulatory Agency before they can be sold in the European Union. In theory, even small subsequent changes in an app (common in nonmedical apps) require recertification. In addition, some companies are concerned about the possibility of being held liable for any harm, which may befall patients using the app. These concerns have led to a reluctance in app developers to go beyond simple monitoring to provide *action plans*. Legislative and regulatory changes may be required to stimulate the development of more intelligent medical apps.

## Appendix

Multimedia Appendix 1 Application features related to the 14 components of the PRISMS taxonomy.

Multimedia Appendix 2 Multiple regression models.

## Abbreviations

BP: blood pressure
DASH: Dietary Approaches to Stop Hypertension
GP: general practitioner
HBP: high blood pressure
HBPM: home blood pressure monitoring
NHS: National Health Services
PRISMS: Practical Reviews In Self-Management Support
cSMS: short message service
