# Supplementary material for: Apps to Support Self-Management for People With Hypertension: Content Analysis
Source: JMIR Mhealth Uhealth. 2019 Jun 3;7(6):e13257. doi: 10.2196/13257 (PMC6746067; doi:10.2196/13257)
Supplement: Multimedia Appendix 2 [file mhealth_v7i6e13257_app2.docx]

**Multimedia appendix 2: Multiple regression models**

| **Outcome variables** | **Predictor variables (coefficient, lower 95% CI, upper 95% CI)** | **Multiple R** | **R Square** | **Adjusted R Square** | **Standard Error** |
| --- | --- | --- | --- | --- | --- |
| Download | *Other possible associated factors:*  Number of raters (0.96,0.91, 1.01)  Rating (0.01, -0.05, 0.06)  Paid (-0.01, -0.06, 0.04)  Apps for multiple conditions ( -0.01, -0.06, 0.04)  Recommended by healthcare professionals/ use in the NHS practices (-0.02, -0.08, 0.03)  Supported by associations or apps for a campaign (-0.001, -0.05, 0.05)  Created by well-known company (-0.01, -0.06, 0.04)  Numbers of features (0.03, -0.02, 0.09) | 0.96 | 0.92 | 0.92 | 0.30 |
| Download | *PRISMS self-management features:*  A. Information about condition and /or its management (0.15, -0.05, 0.36)  B.Information about available resources (-0.03, 0.20, 0.14)  C. Provision of/agreement on specific clinical action plans and/or rescue medication (-0.01, -0.21, 0.19)  D. Regular clinical review (0.02, -0.23, 0.26)  E. Monitoring of condition with feedback (-0.04, -0.28, 0.20)  F. Practical support with adherence (medication or behavioural) (-0.03, -0.23, 0.18)  G. Provision of equipment (0.04, -0.15, 0.22)  H. Provision of easy access to advice or support when needed (-0.04, -0.21, 0.14)  I.Training/rehearsal to communicate with healthcare professionals (-0.03, -0.25, 0.19)  K.Training/rehearsal for practical self-management activities (0.10, -0.10, 0.28)  L.Training/ rehearsal for psychological strategies (-0.04, -0.23, 0.15)  M.Social support (0.22, 0.04, 0.39)  N. Lifestyle advice and support (-0.09, -0.28, 0.11) | 0.28 | 0.08 | -0.02 | 1.01 |
| Average ratings | *PRISMS self-management features:*  A. Information about condition and /or its management (0.12, -0.10, 0.34 )  B.Information about available resources (-0.08, -0.26, 0.10)  C. Provision of/agreement on specific clinical action plans and/or rescue medication (-0.03, -0.24, 0.18)  D. Regular clinical review (-0.01, -0.27, 0.23)  E. Monitoring of condition with feedback (-0.02, -0.26, 0.23)  F. Practical support with adherence (medication or behavioural) (-0.08, -0.29, 0.13)  G. Provision of equipment (-0.20, -0.40, 0.00)  H. Provision of easy access to advice or support when needed (0.04, -0.14, 0.23)  I.Training/rehearsal to communicate with healthcare professionals (0.11, -0.12, 0.34)  K.Training/rehearsal for practical self-management activities (0.08, -0.12, 0.28)  L.Training/ rehearsal for psychological strategies (0.13, -0.07, 0.323)  M.Social support (-0.07, -0.26, 0.11)  N. Lifestyle advice and support (0.06, -0.15, 0.27) | 0.30 | 0.09 | <0.01 | 1.0 |
